# Secondary implant stability in autologous one-stage internal and external sinus floor elevation using resonance frequency analysis – a three-month follow up

**DOI:** 10.1007/s00784-026-07104-2

**Published:** 2026-08-25

**Authors:** Katharina Schaffrath, Kristian Kniha, Mark Ooms, Philipp Winnand, Nils Vohl, Britta Lohn, Frank Hölzle, Ali Modabber

**Affiliations:** 1 Department of Oral and Maxillofacial Surgery, University Hospital RWTH, Aachen, Germany; 2 Department of Oral and Maxillofacial Surgery, University Hospital RWTH Aachen, Pauwelsstraße 30, 52074 Aachen, Germany

**Keywords:** Resonance frequency analysis, Dental implant, Sinus floor elevation, Autologous, One-stage

## Abstract

**Objectives:**

The aim of this retrospective study was to evaluate implant stability using resonance frequency analysis (RFA) (Osstell^®^ W&H, Mettmann) for autologous one-stage sinus floor elevation with implantation and provide advice regarding the healing procedure and the timing of implant loading.

**Materials and methods:**

After 3 months, 145 implants were evaluated for secondary implant stability using RFA. Of the evaluated implants, 72 were set in original bone (OB) in the upper posterior jaw, 21 were set in combination with a one-stage autologous external sinus floor elevation (EE), and 52 in combination with internal elevation (IE).

**Results:**

Osseointegration was significantly higher in OB (94,4%) compared to EE (76,2%) and IE (80,8%). RFA values were best in the OB group (OB: 77,5 [8], EE: 75 [12], IE: 75 [8]; *p* = 0,030).

**Conclusion:**

In general, autologous one-stage sinus floor elevation combined with implantation is a safe procedure, but we recommend a prolonged healing phase of at least four additional weeks for EE and IE procedures.

**Clinical relevance:**

Although one-stage procedures require longer healing phases, treatment duration is still shorter than a two-stage procedure. Patients often prefer minimally invasive and short options because of anxiety and stress.

## Introduction

Primary and secondary implant stability is one of the most important factors for implant survival and successful prosthetic loading. It is important for the dentist to ensure accurate measuring as there are significant differences between these two points [1] and the best time for implant loading often must be determined individually. Therefore, different techniques have been described in the literature. The easiest way to evaluate primary stability is to measure the insertion torque (IT) [2]. Low torque values cause instability and constant micro moving of the implant, while a high IT may be related to very strong bone quality and is inversely correlated with alveolar ridge width [3]. Further, it might be a sign of bone damage due to insufficient preparation and high pressure and, therefore, resulting in the reduced vascularization of the bone surrounding the implant. This decreases the chances of healing. Thus, both low and high torque values may cause implant loss.

Measuring secondary stability is more challenging as the surgeon may use additional techniques such as damping capacity assessment (DCA), where the response on mechanical impact is measured or resonance frequency analysis (RFA), which rates the stiffness of the implant-bone interface. In recent years, many devices have been introduced and tested for applying these techniques. AnyCheck^®^ (MPE Medical, Wesseling) and Periotest^®^ (Medizintechnik Gulden, Modautal) have been used for DCA. Penguin^®^ (Integration Diagnostics Sweden SB, Göteborg) and Osstell^®^ (W&H, Mettmann) have been used for RFA. Although all of these techniques show similar performances [4], sensitivity [5], and reliability, tests [6] indicate differences between the methods and Osstell^®^ showed better reliability [7]. Nevertheless, measurements are comparable in the same system and should not be mixed in the evaluation of an implant [8, 9].

Many factors may influence the stability and survival rate of dental implants. Raz et al. and Merheb et al. found that longer implants in dense bone show a higher primary stability [2, 10]. Kastala et al. show that different implant systems do not influence the stability [11]. Nevertheless, narrow implant diameter and maxillary implants show worse values [12, 13], this includes implantation in older patients, smokers, and patients with a history of periodontitis [14]. But in general, only secondary stability can deliver reliability in implant outcomes [15]. In the literature, there is a lack of information about the survival rates in one-stage implantation combined with autologous sinus floor elevation. Although a mixture of autologous and bovine augmentation material has shown good results [16] and rough surfaces in grafts may optimize the results [17], the stability of one-stage implantation has to be examined.

Therefore, the aim of this study was to evaluate secondary implant stability after three months using RFA (Osstell^®^) when implantation was performed in original bone (OB) and in combination with autologous one-stage external (EE) and internal (IE) sinus floor elevation to evaluate healing periods and adjust recommendations before prosthetic loading of dental implants.

## Materials and methods

**Fig. 1:**
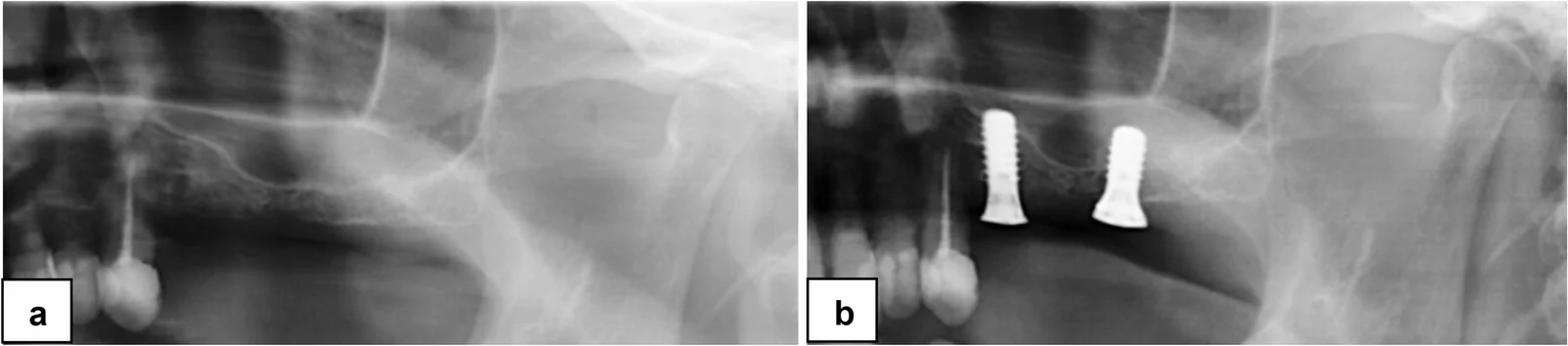
Panoramic x-ray of implantation in the upper leftsided jaw, (**a**) bone height before implantation, (**b**) after implantation (regio 024: one-stage internal elevation, regio 026: one-stage external elevation)

After gaining approval from the ethics committee (EK 25–308), our database was searched for patients who received at least one dental implant in the upper posterior tooth region into OB or in combination with one-stage inlay augmentation—such as internal or external elevation of the maxillary sinus—and measurement of stability with Osstell^®^ (W&H, Mettmann) between January 2024 and June 2025. Exclusion criteria were osteoporosis or antiresorptive therapy in the patient’s history and implantation in combination with two-stage augmentation. Smokers or patients with treated and untreated periodontitis were excluded from this study. To homogenize the group of implants and reduce the statistical influences, zirconia (*n* = 16), bone level (*n* = 12), camlog (*n* = 2) and implants with less than 3,75 mm (*n* = 3) were excluded. 21 implants were excluded because of inappropriate recall of more than 28 days difference to the median of 113 days. In total, 145 implants in 100 patients were included in this study (Straumann^®^ [Basel, Switzerland]). All implants were divided into three groups: implants placed into OB (*n* = 72), implants placed in combination with one-stage external sinus floor elevation (EE: *n* = 21), and implants placed in combination with one-stage internal sinus floor elevation (IE: *n* = 52). An internal procedure was performed in cases where the maximum height to elevate was 3 mm. External elevation with simultaneous implantation was performed with at least 5 mm of OB height (Fig. 1), so primary stability of the implant could be granted. In cases of uncertain implant stability or prognosis, two-stage procedure was performed. Every sinus floor elevation was performed with autologous bone obtained from intraoral sites such as the drill tunnel or via scraping from the zygomatic alveolar crest by two oral surgeons. All implants were primarily stable on the day of the operation, measured by IT of at least 35 N/cm^2^. Implant stability was measured after three months with the Osstell^®^ device. As stated by the manufacturer, a value of less than 60 for implants indicates low stability, while a value between 60 and 69 indicates medium stability. Further, a value greater than 70 shows high stability, and prosthetic loading is recommendable. Although a loading is possible with medium stability, implants were reseased as fully healed and ready for prosthetic loading if a value of at least 70 was measured. If implants were measured under 70, prolonged healing of at least four additional weeks was recommended combined with a new RFA measurement to improve the quality and prognosis of the supply. Implants that had to be removed until the date of measurement were included as RFA value zero.

### Surgical procedure

Following local anesthesia with Ultracain (Sanofi, Frankfurt, Germany) and a short period of exposure, crestal cutting and careful preparation was performed. If necessary, the bone was flattened slightly. After marking the position, the drill tunnel was processed slowly with a rotation speed of up to a maximum of 300 rpm for the first drill. Further, the rotation speed was reduced to 80–100 rpm. Because of the slow rotation, bone fragments can be collected easily, avoiding rinsing them away.

In cases of EE, additional bone was collected from the zygomatic crest and the anterior wall of the sinus using a Safe Scraper (Geistlich, Wolhusen, Swiss). By scraping, the bone thickness can be reduced carefully until the Schneiderian membrane is visible. Care has to be taken to avoid a perforation. The relatively small amount of bone must be placed precisely, which requires a high level of skill. The implant placement follows gold standard protocol with an IT between 35 N/cm^2^ and 50 N/cm^2^. Vicryl 4.0 was used for closure.

### Statistical analysis

The statistical analysis was carried out with Microsoft Excel, IBM SPSS Statistics v. 28 (IBM, New York, USA), and Graph Pad Prism 10. The chi-square test was used to evaluate levels of significance, the Fisher-Freeman-Halton test was used for baseline data, and the Kruskal Wallis test was used for metric data. P values below 0.05 were considered to be statistically significant.

## Results

The medium age of the patients was 62 (IQR: 21) years. In terms of sex, 85 implants belonged to male patients, 60 to female patients. Baseline data did not differ concerning sex and time of measurement, while the patients age was significantly younger in the EE group (Table 1).

**Table 1:** Baseline data

Age (years)	OB (*n* = 72)	EE (*n* = 21)	IE (*n* = 52)	*p* value
	64 (21)	49 (20)	65,5 (22)	0,029
Male	41	12	32	0.867
Female	31	9	20	
Time of measurement (days)	98 (15)	104 (28)	98 (22)	0.398

Data described as median (interquartile range) for patients age and time of measurement and as total amount for sex for OB, EE and IE separately.

In general, the cohort of implants contained titanium tissue level implants. Implants with a length of 8 mm (*n* = 14), 10 mm (*n* = 106), and 12 mm (*n* = 25) and a diameter of 3.75 mm (*n* = 15), 4.1 mm (*n* = 51), 4.8 mm (*n* = 59), and 5.5 mm (*n* = 20) were included. Distribution of different implant lengths (*p* = 0,009) and diameter (*p* = 0,001) differed between the groups. The length of the implants healing phase on the day of the measurement was not significantly different (OB: 98 [15], EE: 104 [28], IE: 98 [22] days; *p* = 0.398).

We recognized a significantly higher implant healing rate in the OB group (94.4%) after three months, while 76.2% for the EE group and 80.8% for the IE group were fully healed and ready for prosthetic loading after three months (Table 2). Implant loss was the highest in the IE group (Table 2), while the time of loss could not be extracted from the data because of uncertain patient statements.

**Table 2:** Implant healing rates measured with Osstell^®^ three months after implantation

	OB (*n* = 72)	EE (*n* = 21)	IE (*n* = 52)	*p* value
Fully healed (RFA > 70)	68 (94.4%)	16 (76.2%)	42(80.8%)	0.033
Not healed yet (RFA < 70)	1 (1.4%)	5 (23,8%)	9 (17.3%)	
Implant loss	3 (4.2%)	0 (0%)	1 (1.9%)	

Data described as total amount (% of group) for OB, EE and IE separately. *P *value for intergroup comparison using Kruskal Wallis test.

**Fig. 2:**
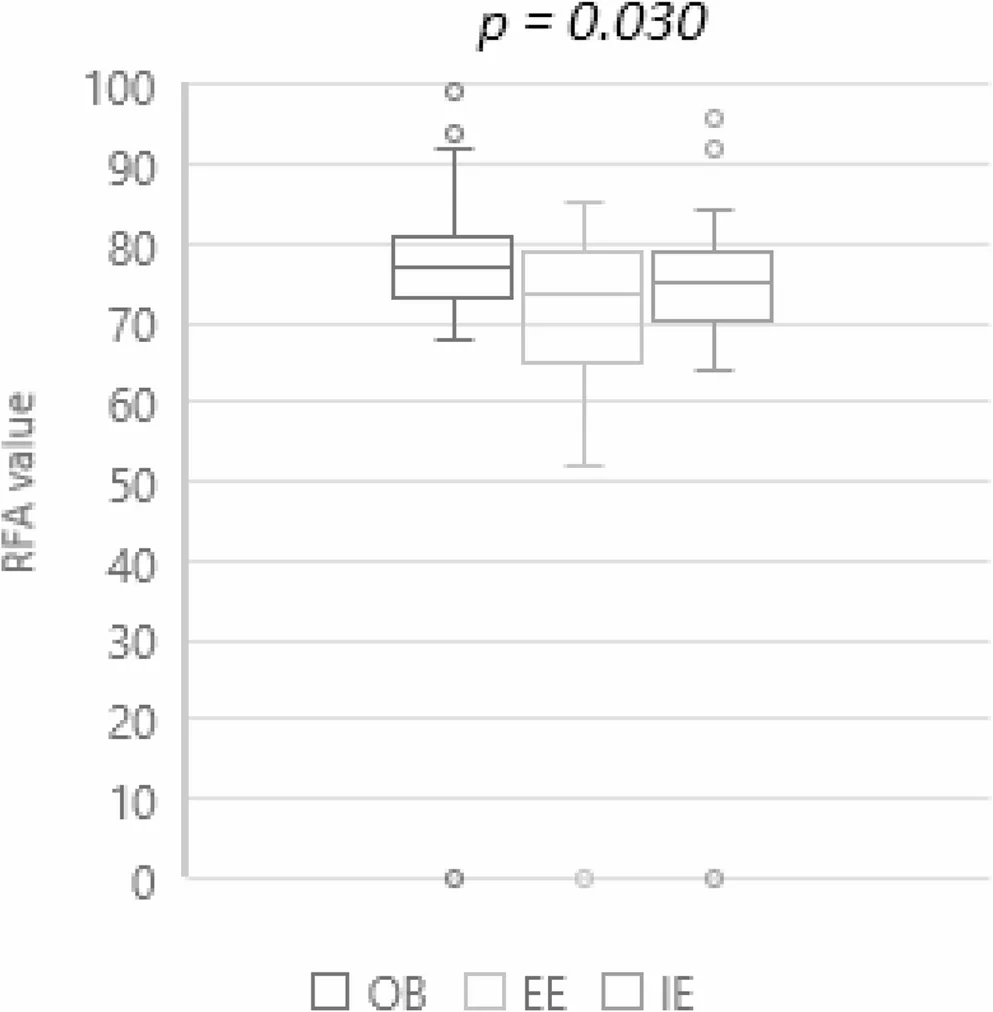
RFA values for OB, EE and IE after three months

RFA values differed between the groups and were the highest in the OB group (Fig. 2). This matches with the data in Table 2. In general, measurements in all three groups were above the minimum fully healed value of 70 (OB: 77.5 [8], EE: 75 [12], IE: 75 [8]; *p* = 0.030).

**Table 3:** Binary logistic regression for OB vs. non-OB implants

Group	Comparison	Odds ratio	*p*-value
	Non-OB vs. OB	3.736	0.036
age	years	1.032	0.078
implant length	mm	0.787	0.523
implant diameter	mm	0.773	0.638

Odds ratios and p-values correspond to a logistic regression model including implant healing as dependent variable and group, age, implant length, and implant diameter as independent variables.

### Measurement after four additional weeks

For 15 implants with a RFA lower than 70 after 3 months, patients were invited after four additional weeks. In 14 of 15 cases the RFA was measured above 70 (fully healed). Taking a closer look only one implant belonging to the IE group was measured 68. Regarding the not yet healed implants a healing rate after four additional weeks of 93,3% was observed.

## Discussion 

Sinus floor elevation often is necessary and is the only option for patients with reduced height of the alveolar crest to receive implants and to get full and fixed dental restoration. This is possible as a one-stage or a two-stage procedure, although healing phases double in the two-stage scenario and immediate prosthetics sometimes are required by the patient, presenting a challenging situation to address.

In this study, we evaluated implants with an RFA value of at least 70 as fully healed, as recommended by the manufacturer. Values between 70 and 80 are also mentioned in the literature [13, 18]. Oh et al. showed even lesser values of about 65 for maxillary implants in OB [13]. These values could be improved by our data for OB and also for autologous one-stage augmentations. Merheb et al. found that longer implants show superior performance in implant stability [10]. This could be supported by our data.

As Pommer et al. reported in their cadaver study, bone density seems to represent the major determinant of primary stability, not only for OB but also for maxillary sinus augmentation [19]. Therefore, it is not only about the bone quality but also about the surgeon’s technique.

The limitations of this study pertain to retrospectivity, as we have group size, diameter, length and age imbalances in our baseline data, which, however, reflects clinical reality. Especially the group size imbalances can be explained by a consecutive enrollment. So in general, the study shows reliable data. RFA is a usable method with excellent repeatability and reproductivity [20] to evaluate the implant status, and it provides a non-invasive mode of assistance to surgeons or dentists to choose the optimal time for implant loading.

The limitations of this study pertain to retrospectivity, as we have group size, diameter, length and age imbalances in our baseline data, which, however, reflects clinical reality. Especially the group size imbalances can be explained by a consecutive enrollment. So in general, the study shows reliable data. RFA is a usable method with excellent repeatability and reproductivity [20] to evaluate the implant status, and it provides a non-invasive mode of assistance to surgeons or dentists to choose the optimal time for implant loading.

Secondary stability is a better indicator for implant survival than primary stability, although Noaman et al. reported that secondary stability can be reduced compared to primary stability [18], or at least show a temporary value drop due to bone rearrangement. In summary, IT is useful for primary stability while RFA should be used to evaluate secondary stability. These techniques and values should not be mixed up or compared with each other.

The challenge with sinus floor augmentation is that primary stability correlates to the remaining OB height. Therefore, indications for one-stage procedures must be set precisely and individually. This study shows that there is a lower healing rate and lower RFA values after three months, so a prolonged healing phase should be recommended and immediate loading should be avoided. Nevertheless, one-stage sinus floor elevation and implantation seems to be a good and safe option with high implant survival rates [21] and low complication rates due to autologous bone graft [22] A clear difference between IE and EE has not been found. This may be due to the fact, that OB height is not the same for every patient. Although surgeries were performed according to standard practices and guidelines, another limitation is that data on the OB height is missing, so the exact differentiation of bone height is not possible. This depicts a significant restriction in the comparison of the IE and EE group, because bone height itself is a crucial factor for implant stability. Although there was primary stability in every single case, which is absolutely necessary for single stage procedures, the higher rate of not yet healed implants in the EE group of 23,8% might be explainable by this fact. Whether RFA values differ depending on the original bone height must be examined in further studies.

Moraschini et al. show that a lack of experience of the surgeon is associated with early implant loss [23]. In our study, all surgeries were performed by two experienced oral surgeons. However, even if implant loss rate is low, questions about the best healing period before loading must be discussed.

In one-stage procedures, autologous bone graft may be the most suitable option. However, in such procedures, the main reason for foreign graft material—the protection from resorption—loses in weight, because the implant itself protects the graft from resorption as it holds as much bone as necessary. As in this study, bone can be harvested from the drilling canal and the maxillary wall by scraping. A second field of operation is not necessary.

Pros such as reduced systemic reactions to foreign materials and reduced costs are already known. Hof et al. show that treatment costs and duration are important for patients as they have high predictability regarding treatment success [24]. Although one-stage procedures require longer healing phases, treatment duration is still shorter than a two-stage procedure. Patients often prefer minimally invasive options because of anxiety and stress. Therefore, this raises the question: Why not use foreign materials as it seems to be an easy and short procedure for the surgeon?

The augmentation with foreign materials, such as xenogenous and alloplastic bone, has important indications, especially if a large amount of material is needed. In many cases this might be the best option if a second field of operation is not desired, either by the patient or by the surgeon, for example, because of systemic factors such as increased risk of anticoagulation. On the other hand, there remains a group of patients who reject foreign materials from another species or another individual for general or religious reasons (e.g., xenogenous materials for Muslim patients).

Therefore, our autologous concept has many pros since we used the bone that we had to remove anyway for grafting. This bone was drilled out of the implant tunnel to avoid a second field of operation, minimizing the risk of infection because we did not use any foreign material. Treatment duration from consultation to implant loading could be reduced compared to a two-stage procedure and costs could be kept low as a second procedure was avoided and we did not use foreign material. Moreover, the surgery was performed by experienced surgeons to improve the probability of sufficient dental healing. Considering that the only tangible con is the longer healing period before dental loading especially for EE procedures, this seems to be a promising concept. Nevertheless, the additional healing time should be examined in further studies as it still depicts gold standard.

All these factors must be discussed with the patient. Autologous one-stage sinus floor elevation is a suitable operative procedure, which can be recommended to an experienced surgeon and a well-informed patient.

## Conclusions 

Autologous one-stage sinus floor elevation combined with implantation is a safe procedure. However, we recommend a prolonged healing phase of at least four additional weeks compared to implantation into OB.

## Data Availability

The datasets used and analyzed during the current study are available from the corresponding author on reasonable request.

## Abbreviations

OB: Implants placed into original bone
EE: Implants placed in combination with one-stage autologous external sinus floor elevation
IE: Implants placed in combination with one-stage autologous internal sinus floor elevation
IT: insertion torque
RFA: resonance frequency analysis
DCA: damping capacity assessment
